# Hepatic Metabolism of Sakuranetin and Its Modulating Effects on Cytochrome P450s and UDP-Glucuronosyltransferases

**DOI:** 10.3390/molecules23071542

**Published:** 2018-06-26

**Authors:** Hyesoo Jeong, Jimin Lee, Soolin Kim, Yoo Yeon Yeo, Hyunyoung So, Honghua Wu, Yun Seon Song, Chang-Young Jang, Hee-Doo Kim, Min Jung Kim, Minsun Chang

**Affiliations:** 1 Graduate School of Biological Sciences, Sookmyung Women’s University, Seoul 04310, Korea; hyesoojeong@sookmyung.ac.kr (H.J.); petia13@naver.com (J.L.); soolinn@sookmyung.ac.kr (S.K.); 2 Department of Biological Sciences and Research Institute of Women’s Health, Sookmyung Women’s University, Seoul 04310, Korea; yooyounyeo@sookmyung.ac.kr (Y.Y.Y.); sohy0131@sookmyung.ac.kr (H.S.); minkim@sookmyung.ac.kr (M.J.K.); 3 Center for Research and Development of Chinese Medicine, Tianjin University of Traditional Chinese Medicine, Tianjin 300193, China; wuhonghua2003@163.com; 4 College of Pharmacy, Sookmyung Women’s University, Seoul 04310, Korea; yssong@sookmyung.ac.kr (Y.S.S.); cyjang@sookmyung.ac.kr (C.-Y.J.); hdkim@sookmyung.ac.kr (H.-D.K.)

**Keywords:** sakuranetin, flavanone, drug metabolism, cytochrome P450, UDP glucuronosyltransferase, drug-herb interaction, metabolic interconversion

## Abstract

Sakuranetin (SKN), found in cherry trees and rice, is a flavanone with various pharmacological activities. It is biosynthesized from naringenin in rice or cherry trees, and the metabolism of SKN has been studied in non-human species. The present study aimed to investigate the metabolic pathways of SKN in human liver microsomes and identify the phase I and phase II metabolites, as well as evaluate the potential for drug–herb interactions through the modulation of drug metabolizing enzymes (DMEs). HPLC-DAD and HPLC-electrospray mass spectrometry were used to study the metabolic stability and identify the metabolites from human liver microsomes incubated with SKN. The potential of SKN to inhibit the DMEs was evaluated by monitoring the formation of a DME-specific product. The cytochrome P450 2B6 and 3A4-inductive effects were studied using promoter reporter assays in human hepatocarcinoma cells. The major pathways for SKN metabolism include B-ring hydroxylation, 5-*O*-demethylation, and conjugation with glutathione or glucuronic acid. The phase I metabolites were identified as naringenin and eriodictyol. SKN was found to be a UDP-glucuronosyltransferases (UGT) 1A9 inhibitor, whereas it induced transactivation of the human pregnane X receptor-mediated cytochrome P450 (CYP) 3A4 gene.

## 1. Introduction

Sakuranetin [(+/−) 7-*O*-methylnaringenin] (SKN) is a member of the flavanone family, and has been reported to possess anti-microbial, anti-inflammatory, anti-mutagenic, anti-leishmanial, and anti-pyranosomal activities [1,2,3,4]. It is produced in various plants through a number of diverse mechanisms, in response to either stress or infection. For example, the synthesis of SKN is induced by ultraviolet light or blast infection in rice (*Oryza sativa*) leaves through the activity of naringenin 7-*O*-methyltransferase (NOMT) [5]. SKN was first identified in the bark of the cherry tree (*Prunus* spp.) as an aglycone of sakuranin, SKN 5-*O*-glucoside (SKNG) [6], and was later found as either SKN or SKNG in many other plants, such as *Artemisia campestris*, *Boesenbergia pandurata*, *Baccharis* spp., *Bertula* spp., *Juglans* spp., and *Rhus* spp.; the extracts of the leaves, stems, or barks of these plants have been used as food, traditional medicine, or herbal supplements, owing to their health-promoting effects [2,7,8,9]. In particular, plants containing SKN and other structurally related flavanones have been used as folk medicines for the treatment of diabetes, inflammatory diseases, allergies, diarrhea, fever, pain, and various cancers [8].

Flavanones are substrates for various drug metabolizing enzymes (DMEs), as well as modulators of these enzymes; hence, flavanones’ bioavailability is heavily dependent on their hepatic metabolic stability, and their inhibition or induction of DMEs may interfere with the metabolism of drugs that are taken concomitantly [10,11]. The potential ability of flavanones to modulate DMEs can result in adverse drug–herb interactions (DHIs), where the pharmacokinetic behavior of a drug is affected by flavanones. For example, naringenin has been identified as being a CYP3A4 inhibitor that inhibits the metabolism of simvastatin (CYP3A4 substrate) [12]. In addition, there is a possibility that metabolic interconversion via various DME-mediated biotransformation steps can lead to the production of a structurally similar class of flavonoid derivatives with distinct pharmacological activities from the parent flavonoid. It has been shown that numerous methoxylated flavonoids can be demethylated to their oxidized analogs [13]. For example, kaempferide is *O*-demethylated to form kaempferol, while tamarixetin is oxidized to form quercetin by human liver microsomes [14,15]. It has been reported that SKN is demethylated to form naringenin or oxidized to form sternbin by rice blast fungus (*Pyricularia oryzae*) or by *Caenorhabditis elegans* [16,17]. However, mammalian pathways involved in the metabolism of SKN, including those involved in its demethylation, have not been reported elsewhere. Considering the increase in scientific evidence-based reports on the pharmacological activities of SKN/SKNG or SKN-containing herbs and their effects on human health, it is important to understand the SKN metabolic pathways and to evaluate the potential for DHIs through the modulation of DMEs.

In the present study, the phase I and II metabolic pathways for SKN were studied using human liver subcellular fractions, and its major metabolites were identified using a HPLC-electrospray (ESI) linear ion trap mass spectrometry (LC-ESI-MS) technique. The potential of SKN to inhibit hepatic microsomal cytochrome P450 (CYP) and UDP-glucuronosyltransferases (UGT) activities, and to induce the transactivation of the CYP2B6 and CYP3A4 genes in a human hepatocarcinoma cell line, was also studied.

## 2. Results

### 2.1. Phase I Metabolism of Sakuranetin

The NADPH-dependent metabolic stability of SKN was determined using liver microsomes prepared from human, mouse, rat, or dog, in order to compare its phase I oxidative metabolism in several species. These four species were chosen for this study because they are frequently used as model species for preclinical Good Laboratory Practice regulatory toxicity studies [18]. After 60 min of incubation, the amount of SKN remaining in the mixture was greater than 50% in the human liver microsomes, and was 4.9%, 29.0%, and 18.5% in liver microsomes from mouse, rat, and dog, respectively (Table 1). Accordingly, the hepatic intrinsic clearance (*CL*_int, hepatic_) values for SKN were much larger than the normal hepatic blood flow for mouse, rat, and dog [19]. These data imply that SKN would be cleared much faster via hepatic phase I metabolism, which is a major route for SKN elimination, in mouse, rat, and dog, compared to human.

### 2.2. Uridine 5′-Diphosphoglucuronic Acid-Dependent Phase II Metabolism of Sakuranetin

The metabolic stability of SKN after UGT-catalyzed conjugation was measured in the presence of both nicotinamide adenine dinucleotide phosphate (NADP^+^) and uridine 5′-diphosphoglucuronic acid (UDPGA) and UDPGA alone. Our study showed that SKN is a good substrate for microsomal UGT in mouse, rat, and dog, since the half-lives of SKN in glucuronide conjugation-mediated metabolism were 3.8, 4.4, and 24.2 min, respectively, and the half-lives of those in oxidative metabolism in conjunction with glucuronide conjugation were 0.9, 2.2, and 5.8 min, respectively (Table 2). SKN is metabolically stable in human liver microsomes, having a half-life of >30 min. The order of metabolic stability was therefore determined to be human > dog > rat > mouse. In liver microsomes of all species, the half-lives of SKN were shorter by more than two-fold when SKN underwent both oxidative and glucuronide conjugative metabolism than with conjugative metabolism alone.

### 2.3. Metabolites of Sakuranetin

**Figure 1:**
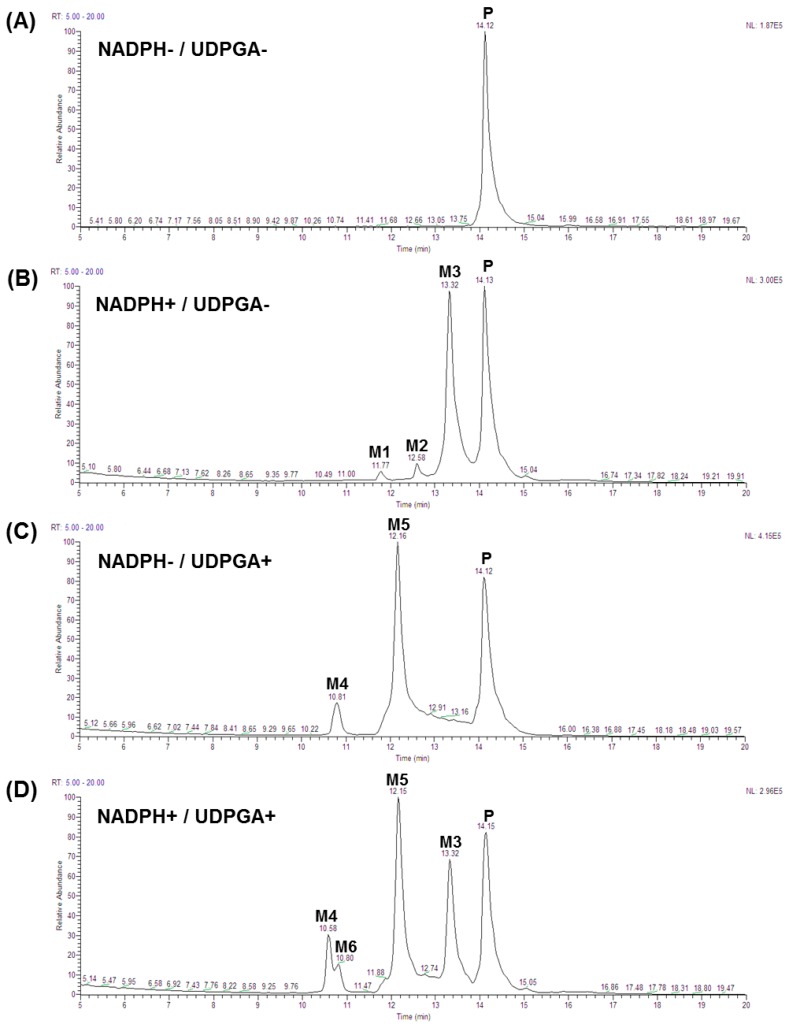
Total ion chromatogram of metabolites of SKN that are dependent on NADPH, UDPGA, or both cofactors. Human liver microsomes were incubated with the appropriate cofactor(s) along with SKN for 60 min. Chromatograms of the incubation mixture with SKN but without cofactors (**A**), NADPH-dependent metabolites (**B**), UPDGA-dependent metabolites (**C**), and the incubation mixture with both NADPH and UPDGA (**D**) are shown. P refers to the parent compound, SKN; whereas the metabolites are designated M1 through M6. The metabolites are identified in Table 3.

**Figure 2:**
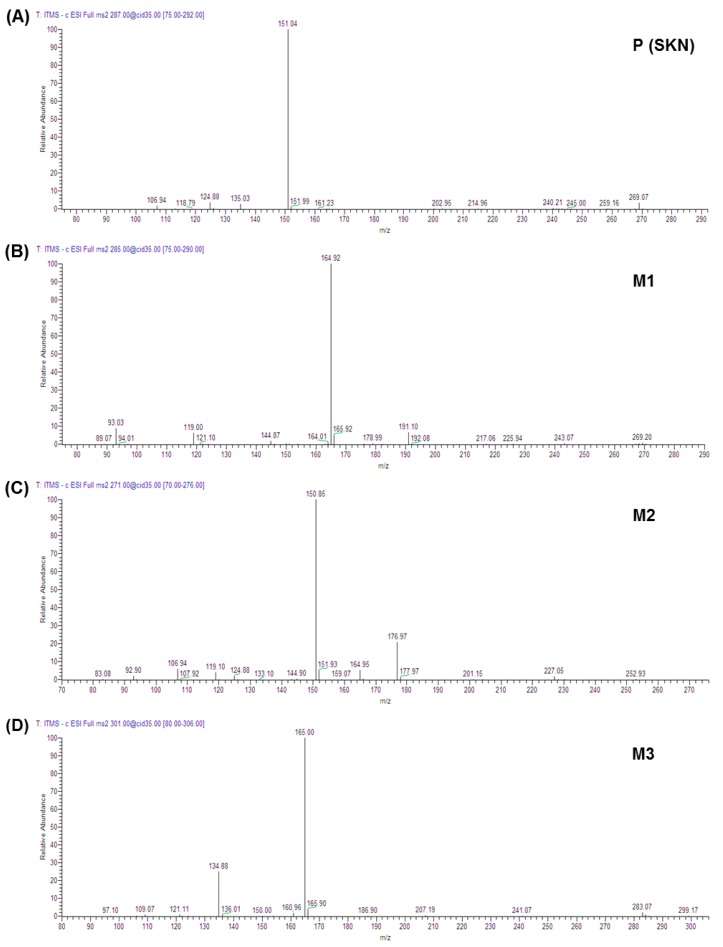

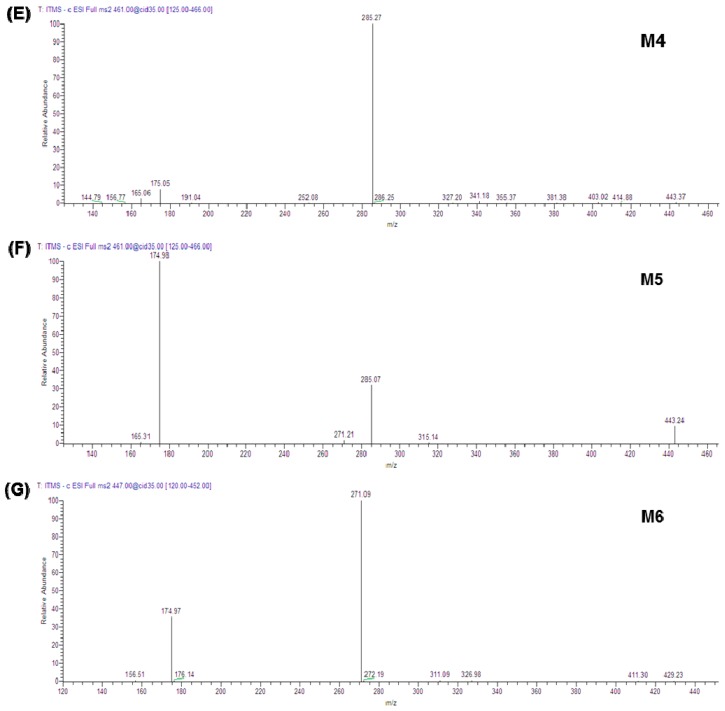
HPLC-electrospray (ESI)-negative mass spectrum of metabolites of SKN dependent on NADPH, UDPGA, or both cofactors. Mass spectrum of SKN (**A**), mass spectrum of M1 (**B**), mass spectrum of M2 (**C**), mass spectrum of M3 (**D**), mass spectrum of M4 (**E**), mass spectrum of M5 (**F**), and mass spectrum of M6 (**G**) are shown. The metabolites are identified in Table 3.

**Figure 3:**
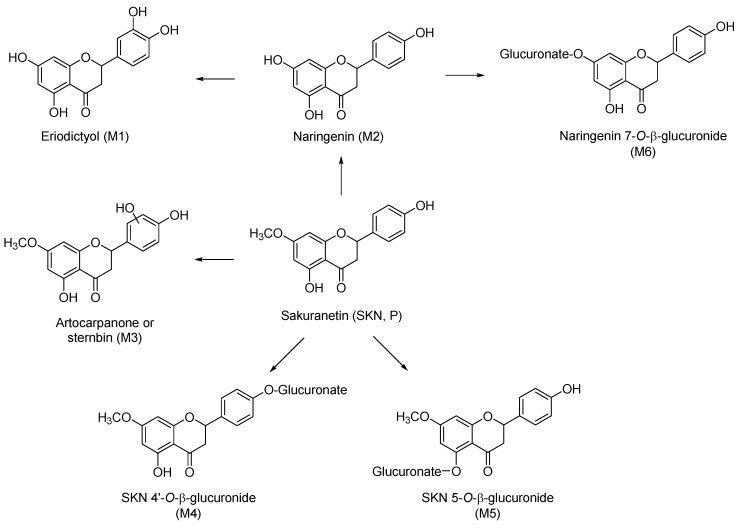
Proposed scheme for the phase I and phase II metabolic pathways of SKN in human liver microsomes. SKN is *O*-demethylated to naringenin (M2), which is then further oxidized to eriodictyol (M1) via flavanone B-ring oxidation. SKN is also oxidized to either artocarpanone or sternbin (M3) by the same type of oxidation. Both SKN and naringenin are subjected to glucuronide conjugation (M4, M5, and M6) by UGT.

The total ion chromatograms and mass spectra for each metabolite are shown in Figure 1 and Figure 2. Fragment ion patterns with the identification of metabolites of SKN are shown in Table 3. CYP-mediated demethylation of the 7-*O*-methyl position of SKN resulted in the metabolic conversion of SKN to naringenin, which is designated M2 and has a retention time of 12.58 min in the total ion chromatogram (Figure 1B and Figure 2C). The identity of M2 was confirmed as naringenin using the standard compound and the Japanese MassBank database (http://www.massbank.jp) (Table 3). Eriodictyol, 3′-hydroxynaringenin (M1), was eluted with a retention time of 11.77 min (Figure 1B and Figure 2B), which was also confirmed using the standard compound and the MassBank database. A mono-oxygenated metabolite of SKN (M3) was an abundant species eluted with a retention time of 13.32 min (Figure 1B,D and Figure 2D). Considering the preference in the oxidative metabolism of naringenin and other flavanones and MassBank of North America (http://mona.fiehnlab.ucdavis.edu), M3 was speculated to be either artocarpanone (2′-hydroxylated SKN) or sternbin (3′-hydroxylated SKN) [16,21]. Glucuronide conjugates with *m*/*z* 461.36 were eluted at retention times of 10.8 and 12.15 min, and each of them was speculated as either SKN 4′-*O*-β-glucuronide (M4) or SKN 5-*O*-β-glucuronide (Figure 1C,D and Figure 2E,F). In addition, the glucuronide formed in the incubation samples with both cofactors was found to be glucuronide of naringenin (M6, Figure 1D and Figure 2G). Based on the retention time and fragment ion patterns, M6 was identified as naringenin 7-*O*-β-glucuronide [22]. Based on the metabolite ID results, the proposed metabolic pathway of SKN is shown in Figure 3.

### 2.4. Inhibition of the Activities of Cytochrome P450 and UDP-Glucuronosyltransferases by Sakuranetin

The inhibitory effects of SKN were examined to monitor the formation of CYP or UGT isozyme-selective metabolites in human liver microsomes in the presence of SKN, using HPLC-DAD or LC-ESI-linear ion trap MS analyses. In order to characterize the mode of inhibition, the enzyme activities of either CYP or UGT were measured, after preincubating the microsomes with SKN for 15 min and then diluting the mixture of microsomes and SKN by 10-fold into enzyme incubation buffer. Pre-incubation of an enzyme with an inhibitor, and subsequent dilution of the enzyme–inhibitor mixture, allows for an examination of whether an inhibitor can cause irreversible change in enzyme activity, in which case the activity would not be restored by dilution. Our data showed that SKN (10 μM) did not show an inhibitory effect greater than 20% on the formation of any CYP isozyme-specific metabolites (Table 4). The activity of UGT1A9 was inhibited by SKN in both co-incubation and pre-incubation samples, with 1.5-fold higher inhibition in the pre-incubation sample than in the co-incubation sample, implying that SKN is an irreversible UGT1A9 inhibitor (Table 5).

### 2.5. Induction of CYP2B6 and CYP3A4 Promoter Activities via CAR and the Pregnane X Receptor

**Figure 4:**
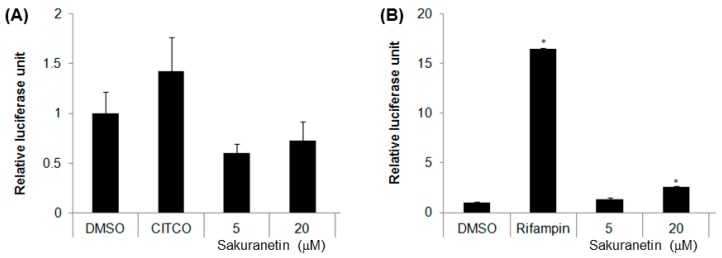
Transactivation of CAR-mediated CYP2B6 (**A**) and PXR-mediated CYP3A4 (**B**) promoter luciferase genes by SKN in HepG2 cells. The expression plasmids coding either hCAR or hPXR were co-transfected into confluent HepG2 cells, along with either the CYP2B6 or CYP3A4-luciferase plasmids. Transfected cells were treated with the appropriate compounds, and luciferase assays were performed. Data are reported as relative luciferase activity unit (RLU: firefly luciferase reading divided by Renilla luciferase reading). Differences among luciferase activities from various treatment groups versus the dimethylsulfoxide (DMSO)-treated group were determined using Student’s *t*-test. An asterisk indicates *p* < 0.001.

Promoter reporter assays were performed to investigate the effects of SKN on the transactivation of CYP2B6 or CYP3A4 genes via a CAR or pregnane X receptor (PXR)-mediated mechanism. In pcDNA3-CAR-transfected HepG2 cells, only CITCO (6-(4-chlorophenyl)imidazo[2,1-*b*] [1,3]thiazole-5-carbaldehyde *O*-3,4-dichlorobenzyl)oxime), a known CYP2B6 inducer, increased luciferase activity (1.5-fold), which was not affected with SKN treatment. These data suggest that treatment of SKN does not induce the binding of hCAR to the CYP2B6 promoter (Figure 4A) in HepG cells. Treatment of HepG cells transfected with pcDNA3-PXR with SKN (20 μM) increased CYP3A4-luciferase activity by 3.8-fold (Figure 4B), implying that SKN could act as CYP3A4 inducer with PXR-associated mechanism.

## 3. Discussion

There is a significant difference between the biological properties of small molecules, such as drugs, xenobiotics, or plant metabolites observed in vitro and those observed in vivo. These differences largely arise from biotransformation, absorption, distribution, excretion, and the bioavailability of small molecules in the two different conditions. Biotransformation serves as a major mechanism for the clearance of chemicals and is a significant cause of the differences in bioactivity and bioavailability seen in in vitro and in vivo assays. In addition, the metabolite profiles and metabolic pathways can provide insight into the pharmacology and toxicology of small molecules. The liver is the main organ responsible for the metabolism of various types of drugs/chemicals; flavonoids are known to be substrates for phase I and II DMEs in the liver [23]. Among flavonoids, flavanones are widely distributed in various plants because they are precursors of all other flavonoid classes [24,25,26]. In particular, flavanones as well as their glycosides are present as the major components in citrus fruits and aromatic plants [26,27]. The bioavailability and biotransformation of flavanones have been most studied using naringenin and its glycoside forms [28,29,30], whereas the pharmacokinetic characteristics of the *O*-methylated naringenin metabolite, SKN, have not been reported elsewhere. In the present study, the metabolic stability and metabolite profiles of SKN were examined to predict the intrinsic hepatic clearance (*CL*_int, hepatic_) and metabolic pathways in the hepatic subcellular fractions. A rapid clearance of SKN via a UDPGA-dependent phase II metabolism was demonstrated in the liver microsomes from mouse, rat, and dog, whereas it was metabolically stable in human liver microsomes, implying that glucuronidation is a major metabolic pathway in mammals with species differences. Xenobiotic compounds with hydroxyl functional groups are known substrates for UGT, which is a major hepatic phase II metabolizing enzyme that catalyzes the formation of *O*-glucuronide conjugates [31]. In addition, NADPH-mediated oxidative metabolism generally involves the ring-hydroxylation of flavonoids, and a hydroxyl group serves as the site for the glucuronide link; thus, oxidative metabolism plays an important role in producing the precursor for the conjugation reaction. Indeed, *CL*_int, hepatic_ increased by more than two-fold in all species of microsomes tested in our study when both NADPH and UDPGA were present in the incubation mixture, compared to when UDPGA alone was present. These data suggest that SKN is susceptible to glucuronide conjugation and NADPH-dependent oxidative metabolism.

The presence of glucuronides (SKN 4′-*O*-β-glucuronide and SKN 5-*O*-β-glucuronide) was confirmed in human liver microsomal incubation samples. Although the flavanoid aglycones are rapidly absorbed, the plasma concentrations of the aglycones are very low, due to extensive first-pass phase II conjugation in the liver, such as glucuronidation, sulfation, or methylation. In particular, hepatic glucuronidation of flavonoids, such as quercetin, naringenin, or hesperetin is known to be one of the most important metabolic pathways, and glucuronides are often the predominant flavonoid forms found in blood or urine [32,33]. The glucuronide metabolites could serve as the reservoir for bioactive aglycones through enterohepatic circulation, resulting in an increased half-life and prolonged pharmacological effects in vivo [34,35,36]. Thus, it can be speculated that glucuronidation of both SKN and its phase I metabolite, naringenin, as demonstrated in the present study, plays a role in the pharmacodynamic and pharmacokinetic behaviors of orally consumed SKN, although further research is required to probe the fate of SKN and its phase I and phase II metabolites in preclinical models.

An important aspect of flavonoid metabolism is that their structural similarities can result in metabolic interconversion. Since metabolism could transform one flavonoid to another, or one class of flavonoid into another, metabolic interconversion might result in a new biological activity. We present here the first report of the metabolic transformation of SKN to naringenin in human liver microsomes. Our results showed that SKN undergoes 7-*O*-demethylation to naringenin, which could then be further metabolized into its phase I and II metabolites, eriodictyol and a glucuronide conjugate, respectively (Figure 3 and Table 3). It is noteworthy that SKN is not produced from naringenin by the mammalian metabolism system, whereas it is produced from naringenin by naringenin *O*-methyltransferase, but only in plants such as rice [5]. The present study demonstrated that eriodictyol was a product of the oxidative metabolism of naringenin by the human liver microsomal enzymes. Naringenin and eriodictyol are flavanones that are abundant in citrus and display antioxidant, phytoestrogenic, cholesterol-lowering, anti-proliferative, as well as CYP-inhibitory activity [12,37,38,39,40]. On the other hand, the efficacy of naringenin and eriodictyol against the growth of cancer cells was reported to be different. It was demonstrated that eriodictyol had a two-fold higher anti-proliferative activity in various cancer cell lines compared to naringenin [41]. In the present study, we assessed if there are differences in biological activity between SKN and its metabolites with respect to inhibitory or inductive effects on DMEs. Our study demonstrated that SKN is a CYP3A4 inducer, whereas naringenin is an inhibitor of CYP1A2, CYP2B6, and CYP3A4, and eriodictyol is an inhibitor of UGT1A1 (Supplementary Materials, Tables S1 and S2). Thus, it can be speculated that modulating activities of SKN on the DMEs may vary, and therefore, differential drug–SKN interaction may occur depending on the metabolic conversion in vivo. In addition, flavanones are potential phytoestrogens, and naringenin has been reported for its phytoestrogenic activity in human breast cancer cells [40]. Our preliminary data show that SKN displays estrogen receptor (ER) agonist-like activity in ER-positive human breast cancer cells (data not shown), implying that it is also able to act as phytoestrogen. Thus, metabolic conversion of SKN to naringenin may lead to prolonged estrogenic effects in vivo.

Emerging data have shown the therapeutic benefits of SKN, indicating that use of SKN-containing preparation would be used as stand-alone or in combination with prescribed drugs. As demonstrated in the present study, SKN is metabolized to glucuronides and is subjected to phase I metabolism, resulting in the formation of other flavanones, which differ in their biological activities and in their ability to inhibit or induce CYPs and UGTs. Although further in vitro, in vivo, and molecular investigations of outcomes of SKN metabolism can provide more compelling evidence regarding the safety profiles of SKN, to the best of our knowledge, our study provides the first report that describes the potential SKN–drug interactions and the metabolic interconversion of SKN in in vitro experimental models.

## 4. Materials and Methods

### 4.1. Chemicals and Reagents

All chemicals and reagents were purchased from Sigma-Aldrich (St. Louis, MO, USA) unless stated otherwise. SKN was purchased from Extrasynthese (Genay, France). Solvents were purchased from Burdick & Jackson (Morristown, NJ, USA). All cell culture reagents were purchased from Gibco (Grand Island, NY, USA) unless stated otherwise. Stock solutions of chemicals were prepared in dimethylsulfoxide (DMSO). Liver microsome or cytosol fractions of human, mouse, and dog were purchased from BD Gentest (San Jose, CA, USA), whereas those of rat were prepared as described previously [42].

### 4.2. Phase I and Phase II Metabolism

Metabolic stability was measured by monitoring the amount of parent compound that remained in an incubation mixture at designated incubation time points. For the phase I metabolism study, SKN (100 μM) was incubated with liver microsomes (1 mg/mL, rat, mouse, and dog; and 0.5 mg/mL, human), and an NADPH-generation cocktail consisting of nicotinamide adenine dinucleotide phosphate (NADP^+^, 1 mM), glucose-6-phosphate (5 mM), glucose-6-phosphate dehydrogenase (1 unit/mL), and MgCl_2_ (10 mM) in potassium phosphate buffer (0.1 M, pH 7.4) in a total volume of 1 mL. To study phase II glucuronide conjugative metabolism, uridine 5′-diphosphoglucuronic acid (UDPGA) (2 mM) was added as a cofactor. The mixture was incubated in a shaking water bath, and the enzyme reaction was initiated by the addition of NADP^+^, UDPGA, or both. Aliquots (100 μL) were removed at 0, 5, 15, 30, 45, and 60 min, and the reaction was terminated by adding two volumes of ice-cold methanol. After centrifugation (12,000× *g*, 4 °C for 15 min) of the reaction mixture, the supernatant (20 μL) was injected into an HPLC-diode array detector (DAD) system to determine the amount of SKN remaining. The half-life of SKN was obtained by plotting the remaining amount of SKN against time using Prism 3.0 software (GraphPad, La Jolla, CA, USA). Hepatic intrinsic clearance (*CL*_int, hepatic_) was calculated using the following formula [20]:

*CL*_int, hepatic_ = (0.693/*t*_1/2_) × (g liver weight/kg body weight) × (mL incubation/mg of microsomal protein) × (mg microsomal protein concentration/g of liver)

For the metabolic pathway study, SKN (100 μM) was incubated with human liver microsomes (0.5 mg/mL), MgCl_2_, and an NADPH-generating cocktail, or UDPGA, or both, in 0.1 M potassium phosphate buffer (pH 7.4) at 37 °C for 60 min in a total volume of 1 mL in a shaking water bath. The reaction was initiated by the addition of a cofactor and terminated by chilling the mixture on ice followed by the addition of an equal volume of ice-cold methanol. Samples were centrifuged for 15 min at 12,000× *g*, and the supernatant was concentrated using a centrifugal vacuum concentrator (Eppendorf AG, Hamburg, Germany). The residue was reconstituted in 30% methanol prior to LC-ESI-MS analysis. Control incubations were performed without a cofactor and showed no evidence of any enzyme-mediated metabolites.

### 4.3. Cytochrome P450 Inhibition Assays

The formation of a metabolite from a CYP isozyme-specific phenotyping reaction (Table 4) was monitored. Each substrate (200 μM), along with SKN (10 μM), was incubated with human liver microsomes (0.5 mg/mL) and an NADPH-generating system in 0.1 M potassium phosphate buffer (pH 7.4) at 37 °C for 60 min in a total volume of 200 μL in a shaking water bath. The metabolic reaction was initiated by adding NADP^+^ (1 mM) and stopped by adding 10 μL of trichloroacetic acid. In some experiments, the microsomes were pre-incubated with SKN for 15 min prior to the addition of the cofactor, in order to determine the time-dependent inhibitory effect. This type of incubation is referred as pre-incubation in Table 4. In contrast, co-incubation refers to experiments in which microsomes and cofactors were incubated in the presence of SKN. After centrifugation of incubation samples at 12,000× *g* for 15 min at 4 °C, the supernatant (20 μL) was injected into an HPLC-DAD system to analyze the metabolites formation (Supplementary Materials).

### 4.4. UDP-Glucuronosyltransferase Inhibition Assays

The formation of a glucuronide conjugate from an isozyme-specific substrate (200 μM, Table 5) was examined to determine UGT activity levels in human liver microsomes. The typical incubation mixture contained human liver microsomes (0.5 mg/mL) along with SKN (10 μM), and MgCl_2_ (10 mM) in 50 mM Tris-HCl buffer (pH 7.4). The metabolic reaction was initiated by adding UPDGA (2 mM) and stopped by adding an equal volume of ice-cold methanol. Either pre-incubation or co-incubation samples were prepared as described in Section 4.3 and shown in Table 5. After centrifugation at 12,000× *g* for 15 min at 4 °C, the supernatant was analyzed by HPLC-DAD for testosterone β-d-glucuronide and LC-ESI-MS for other glucuronides. The conditions used for the operation and analysis of the HPLC-DAD and LC-ESI-MS are provided in the Supplementary Materials and Table S3.

### 4.5. HPLC Instrumentation

The HPLC system (Agilent, Santa Clara, CA, USA) consisted of an Agilent 1200 binary pump, a DAD, and an Agilent 1260 autosampler. A Kinetex^®^ C_18_ reverse-phase column (5 μm, 4.6 × 150 mm) (Phenomenex, Torrance, CA, USA) protected by a KrudKatcher Ultra HPLC in-line filter (Phenomenex) was used for the separation. The mobile phase A was water with 0.1% (*w*/*v*) formic acid, while mobile phase B was acetonitrile. To characterize the remaining amount of SKN present in the incubation mixture, the initial gradient concentration was 10% B and increased to 90% B over 25 min, followed by a return to the initial condition over 5 min. The solvent flow rate was 1 mL/min and the column temperature was maintained at 35 °C. The DAD was set at 280 nm. LC-ESI-MS was performed with an LTQ linear ion trap mass spectrometer (Thermo Scientific, Waltham, MA, USA) with an ESI source, which was coupled with the Agilent HPLC system. The analysis parameters are described in the Supplementary Materials. Mobile phase A consisted of water with 0.1% formic acid, whereas mobile phase B consisted of acetonitrile. The gradient started with 10% B, was increased to 90% B over 15 min, and then was returned to the initial condition over 5 min. The solvent flow rate was set at 1 mL/min, and the column temperature was maintained at 35 °C. The DAD was set at 248 nm for phenacetin *O*-deethylation (PCOD), 298 nm for bupropion hydroxylation (BPHY), 276 nm for diclofenac 4′-hydroxylation (DCHY), 230 nm for tolbutamide 6-hydroxylation (TOLHY), 280 nm for dextromethorphan *O*-demethylation (DEXOD), and 250 nm for testosterone 6β-hydroxylation (TSTHY) and testosterone β-d-glucuronidation (TSTG).

### 4.6. HPLC-Electrospray Mass Spectrometry Analysis

A Kinetex C_18_ reverse-phase column (2.6 μm, 2.1 × 100 mm) (Phenomenex) protected by a KrudKatcher Ultra HPLC in-line filter (Phenomenex) was used for separation. Mobile phase A consisted of water with 0.1% formic acid, whereas mobile phase B consisted of acetonitrile with 0.1% formic acid. The gradient was started at 10% B. It was then increased to 50% B over 5 min and then 90% B over 10 min, followed by a return to the initial condition over 5 min. The solvent flow rate was set at 0.2 mL/min, and the column temperature was set at 35 °C. The LTQ instrument was operated in negative ion electron spray mode at an ion spray voltage of −4.2 kV and a source temperature of 275 °C. The sheath gas flow rate was 35 arb, and the aux gas flow rate was 5 arb. The MS conditions used to detect glucuronides via selected ion monitoring scan mode are summarized in Supplementary Materials Table S3.

### 4.7. Cytochrome P450 Promoter Luciferase Assay

HepG2 cells (American Tissue Culture Collection, Manassas, VA, USA) were maintained in Dulbecco’s modified Eagle’s essential medium (DMEM), containing 10% fetal bovine serum, 1% nonessential amino acids, 1% GlutaMAX^®^, and 1% antibiotics/antimycotics at 37 °C in a 5% CO_2_ atmosphere. The expression plasmid encoding the human constitutive androstane receptor (hCAR; pcDNA3-CAR) and the pGL3-CYP2B6 luciferase construct were kindly provided by Dr. Hongbing Wang (University of Maryland, College Park, MD, USA). The plasmid encoding the human pregnane X receptor (hPXR; pcDNA3-PXR) and the pGL3-CYP3A4 luciferase construct were obtained from Dr. Masahiko Negishi at the National Institutes of Health (Bethesda, MD, USA). Cells were seeded at a density of 1.5 × 10^5^ cells/well into a 24-well plate. The next day, the cells were transfected with 0.25 μg of either pcDNA3-CAR or pcDNA3-PXR and 0.25 μg of a promoter luciferase plasmid (either pGL3-CYP2B6 or pGL3-CYP3A4) using Lipofectamine 2000 (Invitrogen, Carlsbad, CA, USA) according to the manufacturer’s instructions. The transfected cells were grown for 24 h and treated with DMSO (vehicle); CITCO (6-(4-chlorophenyl)imidazo[2,1-*b*] [1,3]thiazole-5-carbaldehyde *O*-3,4-dichlorobenzyl)oxime), a known CYP2B6 inducer (500 nM); rifampin, a known CYP3A4 inducer (10 μM); or flavanones, followed by incubation for an additional 24 h. The cells were harvested and luciferase activity was measured as described previously [43].

### 4.8. Statistical Analysis

Experiments using cells and microsomal incubations were performed in triplicate and repeated at least three times. The results are representative of at least three independent experiments. Statistical comparisons between control and treated groups were performed using a Student’s *t*-test. Differences between data sets were considered statistically significant when the *p* value was less than 0.001. Significant differences are indicated by an asterisk in the figures.

## 5. Conclusions

SKN displays species–specific hepatic microsomal stabilities and has the potential to inhibit or induce CYPs or UGTs. SKN is metabolized to naringenin and eriodictyol in human liver microsomes, suggesting that SKN or the phase I metabolites of SKN would contribute to the modulation of drug metabolizing enzymes, thereby causing the drug–herb interactions.

## Figures and Tables

**Table 1:** NADPH-dependent phase I metabolic stability of sakuranetin (SKN) in liver microsomes from four different species.

Species	Amount Remaining at 30 min (%) ^a^	*t*_1/2_ (min) ^b^	*CL*_int, hepatic_ (mL/min/kg) ^c^
Human	65.5	>60	<20.8
Mouse	4.90	5.40	565
Rat	29.0	10.1	124
Dog	18.5	2.7	641

^a^ Peak area corresponding to SKN in the HPLC-DAD chromatogram compared with that in the 0-min sample chromatogram, which was set as 100%. ^b^ Half-life was obtained using Prism 3.0 (GraphPad, La Jolla, CA, USA) with the assumption that the disappearance of the parent compound with incubation time follows first-order kinetics. ^c^ Values of microsomal protein per liver (MPPL) for human, mouse, rat, and dog were set as 45, 50, 45, and 78, respectively. Liver weight (g)/body weight (kg) values were 20, 88, 40, and 32 for human, mouse, rat, and dog, respectively [20].

**Table 2:** Uridine 5′-diphosphoglucuronic acid (UDPGA)-dependent metabolic stability of sakuranetin (SKN) in liver microsomes from four different species.

UDPGA	+			+		
NADPH	−			+		
Species	Amount Remaining at 30 min (%) ^a^	*t*_1/2_ (min) ^b^	*CL*_int, hepatic_ (mL/min/kg) ^c^	Amount Remaining at 30 min (%) ^a^	*t*_1/2_ (min)	*CL*_int, hepatic_ (mL/min/kg)
Human	71.3	>60	<41.6	54.9	25.0	49.9
Mouse	1.8	3.80	802.4	0.0	0.91	3280
Rat	17.1	4.40	286.8	0.0	2.20	557
Dog	42.4	24.2	71.4	0.0	5.80	300

^a^ Peak area corresponding to SKN in the HPLC-DAD or LC-ESI-MS chromatogram was compared with that in the 0-min sample chromatrogram, which was set as 100%. ^b^ Half-life was obtained using Prism 3.0 (GraphPad, La Jolla, CA, USA), with the assumption that the disappearance of the parent compound with incubation time follows first-order kinetics. ^c^ Values of microsomal protein per liver (MPPL) for human, mouse, rat, and dog were set as 45, 50, 45, and 78, respectively. Liver weight (g)/body weight (kg) values were 20, 88, 40, and 32 for human, mouse, rat, and dog, respectively [20].

**Table 3:** Phase I and phase II metabolites of sakuranetin (SKN) in human liver microsomes.

Label	Precursor Ion, *m/z*	Major Fragment Ions (Relative Abundance)	Identification
Parent phytochemical (Figure 1A)			
P	[M − H]^−^ 285.22	164.92 (100)	SKN ^a^
NADPH-dependent phase I metabolites (Figure 1B and Figure 2B)			
M1	[M − H]^−^ 287.43	151.04 (100)	Eriodictyol ^a^
M2	[M − H]^−^ 271.33	150.85 (100); 176.97 (21)	Naringenin ^a^
M3	[M − H]^−^ 301.41	165.00 (100); 134.88 (25)	Either artocarpanone or sternbin ^b^
Phase II glucuronide conjugation metabolites (Figure 1C and Figure 2E,F)			
M4	[M − H]^−^ 461.52	285.27 (100)	Either SKN-5-*O*-β-glucuronide or SKN-4′-*O*-β-glucuronide ^b^
M5	[M − H]^−^ 461.61	174.98 (100); 285.07 (32); 443.24 (10)	
NADPH and UDPGA-dependent metabolites (Figure 1D and Figure 2G)			
M6	[M − H]^−^ 447.39	271.09 (100); 174.97 (36)	Naringenin 7-*O*-β-glucuronide ^b^

^a^ Identification was performed based on the mass spectra of the authentic standards. ^b^ Identification was performed based on the mass spectra available from the PubChem and MassBank database.

**Table 4:** Cytochrome P450 (CYP) inhibition effects of SKN in human liver microsomes.

CYP Isozyme	Phenotyping Reaction	% Inhibition in Co-Incubation	% Inhibition in Pre-Incubation
1A2	Phenacetin *O*-deethylation (PCOD)	0.4 ± 0.5 ^a^	1.5 ± 2.1
2B6	Bupropion hydroxylation (BPHY)	−0.6 ± 0.9	0.8 ± 0.2
2C9	Diclofenac 4′-hydroxylation (DCHY)	15.3 ± 3.8	8.7 ± 1.4
2C9	Tolbutamide 6-hydroxylation (TOLHY)	2.0 ± 0.6	10.1 ± 1.2
2D6	Dextromethorphan *O*-demethylation (DEXOD)	−0.6 ± 3.1	3.5 ± 2.6
3A4	Testosterone 6β-hydroxylation (TSTHY)	15.6 ± 0.6	12.6 ± 2.8

^a^ Numbers represent % inhibition when metabolite formation in a CYP-mediated reaction without inhibitor was set as 100%.

**Table 5:** UDP-glucuronosyltransferase (UGT) inhibition effects of SKN in human liver microsomes.

UGT Isozyme	Phenotyping Reaction	% Inhibition in Co-Incubation	% Inhibition in Pre-Incubation
1A1	17 β-Estradiol 3-*O*-glucuronidation (ESG)	0.4 ± 0.5	1.5 ± 2.1
1A3	Chenodeoxycholic acid 24-glucuronidation (CDCAG)	−0.6 ± 0.9	0.8 ± 0.2
1A4	Trifluoperazine *N*-glucuronidation (TFPG)	15.3 ± 3.8	8.7 ± 1.4
1A6	1-Naphthol β-d-glucuronidation (NPG)	2.0 ± 0.6	10.1 ± 1.2
1A9	Mycophenolic acid *O*-glucuronidation (MPAG)	−0.6 ± 3.1	3.5 ± 2.6
2B7	Zidovudine 5′-glucuronidation (AZTG)	15.6 ± 0.6	12.6 ± 2.8

## Supplementary Materials

The following are available online: Tables S1–S3.
