# Supplementary material for: Hepatic Metabolism of Sakuranetin and Its Modulating Effects on Cytochrome P450s and UDP-Glucuronosyltransferases
Source: Molecules. 2018 Jun 26;23(7):1542. doi: 10.3390/molecules23071542 (PMC6100415; doi:10.3390/molecules23071542)
Supplement: Supplementary file 1 [file molecules-23-01542-s001.pdf]

Hepatic Metabolism of Sakuranetin and Its Modulating Effects on Cytochrome P450s and UDP-Glucuronosyltransferases

Hyesoo Jeong[1], Jimin Lee[1], Soolin Kim[1], Yoo Yeon Yeo[2], Hyunyoung So[2], Honghua Wu[3], Yun Seon Song[4], Chang Young Jang[4], Hee-Doo Kim[4], Min Jung Kim[2], and Minsun Chang[2],*

[1] Graduate School of Biological Sciences, Sookmyung Women's University, Seoul, Republic of Korea; hyesoojeong@sookmyung.ac.kr (H.J.); petia13@naver.com (J.L.); soolinn@sookmyung.ac.kr (S.K.)

[2] Department of Biological Sciences, Sookmyung Women's University, Seoul 04310, Republic of Korea; yooyounyeo@sookmyung.ac.kr (Y.Y.Y.); sohy0131@sookmyung.ac.kr (H.S.); minking@sookmyung.ac.kr (M.J.K.)

[3] Center for Research and Development of Chinese MedicineTianjin University of Traditional Chinese Medicine, Tianjin, China; wuhonghua2003@163.com

[4] College of Pharmacy, Sookmyung Women's University, Seoul 04310, Republic of Korea; yssong@sookmyung.ac.kr (Y.S.S.); cyjang@sookmyung.ac.kr (C.Y.J.); hdkim@sookmyung.ac.kr (H.D.K.)

* Correspondence: minsunchang@sookmyung.ac.kr; Tel.: +82-2-2077-7626

Supplementary Materials:

Table S1. CYP inhibition effects of naringenin and eriodictyol in human liver microsomes.

CYP isozyme	Phenotyping reaction	Inhibition (%)	
		Naringenin[a]	Eriodictyol[b]
1A2	Phenacetin *O*-deethylation (PCOD)	35.7 ± 2.6[c]	15.9 ± 1.8
2B6	Bupropion hydroxylation (BPHY)	24.0 ± 2.2	16.1 ± 0.9
2C9	Diclofenac 4'-hydroxylation (DCHY)	9.1 ± 1.8	1.0 ± 0.7
2C9	Tolbutamide 6-hydroxylation (TOLHY)	12.0 ± 0.7	7.7 ± 0.4
2D6	Dextromethorphan *O*-demethylation (DEXOD)	11.9 ± 1.4	5.6 ± 3.6
3A4	Testosterone 6β-hydroxylation (TSTHY)	39.6 ± 2.2	3.2 ± 0.7

[a,b] % Inhibition was measured from incubation samples which includes human liver microsomes (0.5 mg/mL), a cofactor-generating system, and either naringenin (a, 10 μM) or eriodictyol (b, 10 μM) as an inhibitor. [c]Numbers represent the % inhibition. Metabolite formation in a CYP-mediated reaction in the absence of an inhibitor was set as 100%.

Table S2. UGT inhibition effects of naringenin and eriodictyol in human liver microsomes.

UGT isozyme	Phenotyping reaction	Inhibition (%)	
		**Naringenin[a]**	**Eriodictyol[b]**
1A1	β-Estradiol 3-*O*-glucuronidation (ESG)	10.8 ± 3.0c	20.8 ± 3.4
1A3	Chenodeoxycholic acid 24-glucuronidation (CDCAG)	2.0 ± 0.9	15.7± 2.9
1A4	Trifluoperazine *N*-glucuronidation (TFPG)	9.6 ± 3.1	13.7 ± 3.8
1A6	1-Naphthol β-D-glucuronidation (NPG)	4.1± 3.1	1.9 ± 2.7
1A9	Mycophenolic acid *O*-glucuronidation (MPAG)	2.0 ± 2.1	2.9 ± 0.8
2B7	Zidovudine 5'-glucuronidation (AZTG)	6.0 ± 2.2	8.2 ± 1.4

[a,b] % Inhibition was measured from incubation samples which includes human liver microsomes (0.5 mg/mL), a cofactor-generating system, and either naringenin (a, 10 μM) or eriodictyol (b, 10 μM) as an inhibitor. [c]Numbers represent the % inhibition. Metabolite formation in a UGT-mediated reaction in the absence of an inhibitor was set as 100%.

Table S3. Selected ion monitoring parameters for quantification of glucuronides by HPLC-MS/MS

Parameters.	1A1[a]	1A3	1A4	1A6	1A7	2B7
Detection mode	Negative	Negative	Positive	Negative	Negative	Negative
Capillary voltage	-50	-10	41	-41	-38	-43
Tube lens	-127.5	-32.5	170.0	-107.5	-102.5	-97.5
Analyte *m*/*z* transition	447→271	567→391	584→408	319→112	495→391	442→125
Retention time	9.5	10.9	11.3	9.5	10.2	10.9

[a] %The type of phenotyping reaction is referred from Table S2.
